# Ligation of the Jugular Veins Does Not Result in Brain Inflammation or Demyelination in Mice

**DOI:** 10.1371/journal.pone.0033671

**Published:** 2012-03-21

**Authors:** Wendy Atkinson, Reza Forghani, Gregory R. Wojtkiewicz, Benjamin Pulli, Yoshiko Iwamoto, Takuya Ueno, Peter Waterman, Jessica Truelove, Rahmi Oklu, John W. Chen

**Affiliations:** 1 Center for Systems Biology, Massachusetts General Hospital and Harvard Medical School, Richard B. Simches Research Center, Boston, Massachusetts, United States of America; 2 Division of Neuroradiology, Department of Radiology, Massachusetts General Hospital and Harvard Medical School, Boston, Massachusetts, United States of America; 3 Division of Vascular Imaging and Intervention, Department of Radiology, Massachusetts General Hospital and Harvard Medical School, Boston, Massachusetts, United States of America; 4 Sir Mortimer B. Davis Jewish General Hospital and McGill University, Montréal, Quebec, Canada; Kyushu University, Japan

## Abstract

An alternative hypothesis has been proposed implicating chronic cerebrospinal venous insufficiency (CCSVI) as a potential cause of multiple sclerosis (MS). We aimed to evaluate the validity of this hypothesis in a controlled animal model. Animal experiments were approved by the institutional animal care committee. The jugular veins in SJL mice were ligated bilaterally (n = 20), and the mice were observed for up to six months after ligation. Sham-operated mice (n = 15) and mice induced with experimental autoimmune encephalomyelitis (n = 8) were used as negative and positive controls, respectively. The animals were evaluated using CT venography and ^99m^Tc-exametazime to assess for structural and hemodynamic changes. Imaging was performed to evaluate for signs of blood-brain barrier (BBB) breakdown and neuroinflammation. Flow cytometry and histopathology were performed to assess inflammatory cell populations and demyelination. There were both structural changes (stenosis, collaterals) in the jugular venous drainage and hemodynamic disturbances in the brain on Tc99m-exametazime scintigraphy (p = 0.024). In the JVL mice, gadolinium MRI and immunofluorescence imaging for barrier molecules did not reveal evidence of BBB breakdown (*p* = 0.58). Myeloperoxidase, matrix metalloproteinase, and protease molecular imaging did not reveal signs of increased neuroinflammation (all *p*>0.05). Flow cytometry and histopathology also did not reveal increase in inflammatory cell infiltration or population shifts. No evidence of demyelination was found, and the mice remained without clinical signs. Despite the structural and hemodynamic changes, we did not identify changes in the BBB permeability, neuroinflammation, demyelination, or clinical signs in the JVL group compared to the sham group. Therefore, our murine model does not support CCSVI as a cause of demyelinating diseases such as multiple sclerosis.

## Introduction

Multiple sclerosis (MS) is a chronic, inflammatory disease of the central nervous system that affects over two million people worldwide [1], [2], [3], [4]. It is characterized by blood-brain barrier breakdown, neuroinflammation, and demyelinating plaques. However, the exact pathogenesis of MS is still unknown and there is currently no known cure. Recently, a new hypothesis implicating chronic cerebral venous insufficiency, coined chronic cerebrospinal venous insufficiency (CCSVI), as the cause of MS has gained widespread attention and interest from patients and physicians [5], [6]. There is an increasing number of studies linking cerebral venous insufficiency to various neurologic diseases such as transient global amnesia [7] and MS [6]. The idea of MS having a vascular etiology was first introduced over a century ago by Charcot who found thickening of small blood vessels in MS patients [8], but other hypotheses (autoimmune, toxin, and virus) are more commonly invoked as possible causes.

First described by Zamboni and co-workers, CCSVI is characterized by impaired extracranial venous drainage, notably in the internal jugular veins (IJVs), vertebral veins (VVs), and deep cerebral veins (DCVs), and the formation of collateral vessels [6]. It has been proposed that CCSVI may lead to increased iron deposition in the brain, leading to an inflammatory or immune reaction and the formation of MS lesions [5], [6], [9], [10], [11]. Percutaneous transluminal angioplasty and stenting have emerged as potential treatment options for MS patients as a result of this new hypothesis [12]. However, the invasive nature of the procedure can lead to serious complications, including stent migration, cerebral hemorrhage, jugular vein thrombosis, and even death [13]. Since the original study on CCSVI and its reported strong association to MS, several other studies have not been able to show a definitive confirmation of the CCSVI hypothesis for MS [14].

Given that there is no cure for MS and the current disease modifying immunomodulatory therapies only help to slow the disease progression, treatment of CCSVI could represent a breakthrough for MS patients if CCSVI is indeed the cause of MS. Conversely, MS patients may be at risk from unnecessary endovenous interventions if the validity of the CCSVI hypothesis is ultimately disproven. To date, there has been no animal model of CCSVI reported in the literature that investigates the relationship between venous congestion arising from extracranial stenosis and the development of demyelination. Thus, we aimed to investigate this hypothesis in a controlled animal model by creating chronic cerebral venous insufficiency in mice. We hypothesized that these animals could exhibit the hallmarks of demyelinating diseases such as MS, including clinical signs, blood-brain barrier (BBB) breakdown, neuroinflammation, and demyelination.

## Materials and Methods

### Ethics statement

Ethical approval was obtained from the Institutional Animal Care and use Committee of Massachusetts General Hospital (Animal Welfare Assurance #A3596-01) in accordance with national and international guidelines. Massachusetts General Hospital maintains full accreditation from the Association for Assessment and Accreditation of Laboratory Animal Care. All experimental procedures were performed only with sedated animals and appropriate analgesia was administered. Animal welfare and steps were taken to ameliorate suffering in accordance with the recommendations of the Weatherall report (2006).

### Jugular vein ligation (JVL)

Forty-three mice were used for the study. Surgical ligation of the right and left JVs was performed on female SJL mice (n = 20) 8–12 weeks old (NCI-Frederick, Frederick, MD). Sham surgery involving exposure of the JVs but no ligation was performed on age and sex-matched SJL mice (n = 15). Experimental autoimmune encephalomyelitis (EAE) was also induced in SJL mice as a positive control (n = 8). The SJL strain of mice was chosen because it has been shown to have increased susceptibility to developing inflammatory demyelination [15] seen in MS.

### EAE induction

Female SJL mice 8–12 weeks old (NCI-Frederick, Frederick, MD) were used with synthetic proteolipid protein (PLP139-151, Anaspec, Fremont, CA) to induce EAE. One milligram of PLP and 4 mg of M. tuberculosis H37Ra (Difco, Detroit, MI) were dissolved in 0.5 ml of completed Freud's adjuvant (Sigma-Aldrich, St. Louis, MO). The content was emulsified using a high-speed homogenizer for approximately two minutes on ice. Each mouse received 100 µl of the PLP emulsion (25 µl each in the inguinal and axillary regions bilaterally). On days 0 and 2, 0.1 µg of pertussingen (P7208, Sigma-Aldrich, St.Louis, MO) dissolved in 200 µl of phosphate-buffered solution was injected intravenously via the tail vein.

### Evaluation of neurological disease

The mice were monitored and weighed at least three times a week, and were followed clinically using the standard 5-point EAE staging system for 4–6 months post-surgery: 0 = no signs, 1 = complete tail limpness without limb weakness, 2 = limb weakness without obvious paralysis on ambulation, 3 = one limb with partial paralysis, 4 = one limb with complete paralysis, and 5 = moribund [16].

### CT venogram and 3D reconstruction

CT venogram was performed on an animal CT scanner (Siemens, Washington DC) in a subset of mice to confirm ligation of JVs in the JVL group (n = 6) and JV patency in the sham group (n = 3) at four months post-surgery to identify chronic structural changes resulting from JV ligation. CT acquisition consisted of 360 cone beam x-ray projections over 360 degrees with a moving source fixed to a slip-ring gantry. X-ray source power and current were 80 keV and 500uA, respectively. Projections were then reconstructed into three-dimensional isotropic volumes containing 512×512×768 voxels, and a voxel dimension of 0.110×0.110×0.110 mm. During CT, mice were continuously injected with an iodine contrast agent (Isovue-370, Bracco Diagnostics, Princeton, NJ) at 20 uL/min for the 10-minute scan. 3D reconstruction was performed on the contrast enhanced CT images using the Amira software (San Diego, CA) by first segmenting out the bone via thresholding, deleting the bone region-of-interest (ROIs), and lastly, using region based thresholding on the unsegmented region to segment out the vessels.

### Magnetic resonance imaging

All chemicals were obtained from Sigma-Aldrich (St. Louis, MO) except where indicated. DTPA-Gd (Magnevist) was obtained from Berlex (Mountville, NJ). The myeloperoxidase (MPO)-sensing MR imaging agent bis-5HT-DTPA(Gd) (MPO-Gd) was synthesized as previously described [17], [18]. Briefly, 100 mg (0.28 mmol) of DTPA-bisanhydride was reacted with serotonin (Alfa Aesar, Ward Hill, MA) in dimethylformamide (DMF [4 mL]) in the presence of 100 µL (1 mmol) of pyridine and 50 mg (2.8 mmol) of ascorbic acid. The mixture was then stirred for 30 minutes at room temperature. The crude product was subsequently precipitated with ether, dissolved in water and passed through a C18 cartridge with water and acetonitrile mixtures. The synthesis of corresponding Gd^3+^ complexes was performed in a 5% citric acid solution (pH = 5.0) at room temperature. GdCl_3_•6H_2_O was used in excess (1.5-fold) in comparison to the chelates and the reaction was stirred for 1 hour. The final product was purified using high-performance liquid chromatography and peaks were detected at 280 nm. The purity of the agent was confirmed by mass spectrometry.

To evaluate whether our model causes cerebral inflammation, we performed MPO-Gd molecular imaging to assess in vivo MPO activity. MPO is a highly oxidizing enzyme secreted in abundance by activated neutrophils, macrophages, and microglia in inflammation, and MPO-Gd is an activatable MR imaging agent that can report MPO activity in vivo with high specificity and sensitivity [16], [19], [20], [21]. MRI was performed on a subset of JVL mice (n = 12) and sham mice (n = 4) between 4–6 months post-surgery. Four of the JVL mice were also imaged at one month after surgery. Additionally, we also imaged EAE mice (n = 3) at peak acute disease (day 10). One JVL mouse was found to have a small subdural hematoma, and was excluded from the study because subdural hematoma is not a reported feature or outcome of CCSVI or MS. Imaging was performed using a dedicated mouse brain coil (model T8118) on an animal 4.7 T MRI scanner (Bruker) consisting of T2- (TE = 20.0 ms, effective TE = 60.0 ms, TR = 2460 ms, 8 averages) and T1- (TE = 8.480 ms, TR = 873 ms, 14 averages) weighted scans before and after intravenous administration of MPO-Gd (0.3 mmol/kg). Post contrast T1 images were obtained every 15 minutes for 60 minutes. Respiratory gating was used during all scans. DTPA-Gd (0.3 mmol/kg) was imaged using a similar protocol as above except only one post contrast T1 time point was acquired.

The images were analyzed using a house-built Matlab program, which creates an MPO activity map of the brain. ROI's were manually created using the Amira software for the muscle background and noise (outside of mouse image) and the brain was automatically segmented in a Matlab script. These ROI's were then used to calculate the activation ratio [21] on a voxel-by-voxel basis utilizing muscle as background instead of normal-appearing brain, which may contain subtle inflammation.

The MRI T1 gadolinium enhanced images were performed to assess contrast agent extravasation as a marker for blood-brain barrier (BBB) breakdown. Images were quantified by manually segmenting out an ROI of normal brain and an area outside the boundaries of the mouse for noise. An automatic segmentation algorithm was used to segment out the brain in utilizing Matlab, which finds the largest connected region in the head. Contrast-to-noise ratios of the post-Gd images was found by taking the mean value over the whole brain minus the mean normal brain background and dividing by the standard deviation of the noise.

### Nuclear scintigraphy

Some mice in the JVL group (n = 4) and sham group (n = 4) were injected with approximately 250 uCi of technetium-99 m (half life = 6.03 hours) labeled exametazime (Ceretec, GE Healthcare, Waltham, MA.) at 4 months post-surgery to assess hemodynamic changes from the JVL. Thirty minutes after injection, the mice were sacrificed, perfused with 20 ml of ice-cold saline, and the brain was excised and the radioactivity counted on a Wallac Wizard 1480 automatic gamma counter (Perkin-Elmer, Waltham, MA).

The gamma counter data was decay corrected back to the time of animal sacrifice. The percent injected dose per gram of tissue (%IDGT) was calculated for each brain, representing the measured tissue scintigraphy count as a percentage of amount (gram) of brain tissue and injected dose (i.e. normalized to correct for tissue mass and injected dose).

### Fluorescence molecular tomography (FMT)-CT Imaging

We also performed FMT-CT in a subset of mice at 6 months of age (n = 3 sham, n = 3 JVL mice) to assess whether matrix metalloproteinase (MMP) and protease activities, which are markers for BBB breakdown and inflammation [22], [23], are elevated in the JVL mice. Twenty-four hours prior to imaging mice received an intravenous injection of 2 fluorescent imaging agents (MMPSense680 and ProSense750, 2 nmol per agent, PerkinElmer, Waltham, MA). MMPSense680 is an activatable fluorescent probe that can detect matrix metalloproteinase (MMP) activity, in particular MMP-2, -3, -9, and -13. ProSense750 is a protease activatable fluorescent probe that can detect cathepsin B, L, S, and plasmin activity. Anesthetized mice were depilated prior to imaging. Mice were placed in the imaging cassette, which has been designed with built-in fiducial markers (four holes filled with gel water for CT and FMT to detect) for enhanced post-processing image fusion [24]. FMT imaging was carried out on a commercial FMT2500 system (PerkinElmer, Waltham MA). Total fluorescent signal, expressed in picomole scale, were determined by placing regions of interest over the mouse brain in the FMT-CT fused reconstructed scans.

Mice were then transferred to the CT scanner (Siemens, Washington DC) in the cassette to undergo anatomical imaging for spatial localization of the FMT signal. CT acquisition was performed similarly as above for CT venogram.

Utilizing the fiducial markers on the mouse cassette, FMT and CT datasets were fused to produce images showing both fluorescent activity and anatomical structure. Coronal CT images were point-based co-registered to the FMT datasets using OsiriX.

### Isolation of brain inflammatory cells and flow cytometry

#### Inflammatory cell isolation

Mice were transcardially perfused with 20 ml of ice-cold phosphate-buffered saline (PBS). Extracted brains were stored in ice-cold Hanks' balanced salt solution (HBSS), containing 15 mM HEPES (N-2-hydroxyethylpiperazine-N′-2-ethanesulfonic acid) and 0.5% glucose. Brain tissue was mechanically dissociated in a loose-fitting glass tissue homogenizer in 5 ml of ice-cold HBSS. After homogenization, the cell suspension was filtered through a 40-µm cell strainer (BD Bioscience, San Jose, CA) into 50 ml conical tubes, and washed with 15 ml ice-cold HBSS. Finally, cells were pelleted at 500× g for 10 min at 4°C, the supernatant was discarded, and cells were resuspended in 10 ml 30% Percoll (17-0891-01, GE Healthcare, Boston, MA) at room temperature. The resultant cell suspension was gently overlaid over 3 ml 70% Percoll in a 15 ml conical tube, so that a sharp interface between the two layers was visible [25]. The density gradient was then centrifuged in a swinging bucket rotor at 800× g for 25 min at room temperature, without brakes applied. After centrifugation, a thick myelin-containing layer at the top was removed and the cells at the 30/70 interface were collected. The suspension was then diluted at least threefold with ice-cold Dulbecco's PBS (DPBS), centrifuged at 500× *g* for 10 min at 4°C, and resuspended using 1 ml staining buffer (1% FBS and 0.5% BSA in DPBS). The cells were then counted and stained for flow cytometry.

#### Flow cytometry

The following antibodies (all from BD Bioscience unless stated otherwise) were used: anti-CD90-PE, 53-2.1; anti-NK1.1-PE, PK136; Anti B220-PE, RA3-6B2; anti-CD49b-PE, DX5; anti-Ly-6G-PE, IA8; anti-CD45.1-APC, A20; anti-CD11b-APC-Cy7, M1/70; anti-F4/80-Biotin, C1:A3-1 (BioLegend, San Diego, CA); and anti-CD11c-Biotin, HL3). Streptavidin-PerCP was used to label biotinylated antibodies. Lineage antibodies (Lin), consisting of CD90, NK1.1, B220, CD49b, and Ly-6G were chosen based on work performed by Swirski and colleagues [26]. CD90 was used as a pan-T-cell marker, NK1.1 for natural killer cells, B220 for B-cells, CD49b for any remaining natural killer or T-cells not positive for CD90, and Ly-6G as a specific marker for granulocytes. The importance for CD49b in EAE has been shown in a recent study [27].

Doublets were excluded on an SSC-height versus SSC-width plot, and all leukocytes were then identified as CD45.1+. Myeloid cells were identified as CD11b^+^ Lin^−^ cells. Neutrophils were CD11b^+^ Lin^+^, and Lymphocytes were CD11b^−^ Lin^+^. Myeloid cells were further divided into macrophages/microglia (F4/80^high^ CD11c^±^) and monocytes (F4/80^−/low^ CD11c^−^). Although there are early reports of F4/80 positive monocytes in the literature, flow cytometry can differentiate between high expression of F4/80 in macrophages/microglia versus low or even absent expression in monocytes. This was recently demonstrated for monocytes from spleen, blood, and bone marrow [26]. The total number of cells was determined under a microscope with a hemocytometer (Reichert, Buffalo, NY) according to manufacturer's instructions. Data were acquired on an LSRII (BD Biosciences) and analyzed with FlowJo 9.3.2 (Tree Star, Inc., Ashland, OR).

### Histopathological analysis

Following MR imaging, mice were sacrificed and the brains were harvested for histopathological analysis. The brains were embedded in optimal cutting temperature (O.C.T) compound (Sakura Finetek, Torrance, CA) with isopentane on dry ice. Serial 6 micron thick fresh-frozen sections were prepared for histopathological analysis. All the histological images were captured using a digital slide scanner, NanoZoomer 2.0RS (Hamamatsu, Japan).

#### Immunohistochemical analysis

Brain tissue sections were briefly treated with 0.3% hydrogen peroxidase solution prior to incubating with rat anti-mouse Mac-3 antibody (BD Biosciences, San Diego, CA) and rabbit polyclonal myeloperoxidase (MPO) antibody (Ab-1; NeoMarkers, Fremont, CA). Biotinylated secondary antibodies (biotinylated anti-rat IgG and biotinylated anti-rabbit IgG)(Vector Laboratories, Burlingame, CA) were applied respectively, and an avidin-biotin complex (ABC) kit (Vector Laboratories, Burlingame, CA) and a 3-amino-9-ethylcarbazole (AEC) substrate (DakoCytomation, Carpinteria, CA), which produces a red end-product in the presence of peroxidase, were used for the color development. All sections were counterstained with Harris hematoxylin solution and a coverslip was applied using an aqueous mounting medium.

#### Detection of demyelination

Luxol fast blue (LFB) staining was performed using the luxol fast blue-cresyl echt violet stain kit (DBS). The brain sections were incubated in luxol fast blue stain solution at room temperature for 24 hours and differentiated in 0.05% lithium carbonate solution, followed by 70% ethyl alcohol until gray and white matter was clearly defined. The sections were counterstained with 0.1% cresyl echt violet stain solution, dehydrated, and mounted with a permanent mounting medium.

#### Immunofluorescence microscopy

Fresh-frozen brain sections (adjacent sections from immunohistochemistry) were incubated with rat anti-mouse CD31 antibody (PECAM-1, clone MEC13.3, BD Biosciences, San Diego, CA), and either rabbit polyclonal occludin antibody (Abcam, Cambridge, MA) or rabbit polyclonal claudin-5 antibody (Abcam, Cambridge, MA). Appropriate biotinylated secondary antibodies followed by streptavidin-FITC (Vector Laboratories, Burlingame, CA) for CD31, and streptavidin-DyLight594 (Vector Laboratories, Burlingame, CA) for occludin and claudin-5 were applied. The slides were coverslipped using a mounting medium with DAPI (Vector Laboratories, Burlingame, CA) to identify the nuclei. Images were captured and processed using an epifluorescence microscope, Nikon Eclipse 80i (Nikon Instruments, Melville, NY), with a Cascade Model 512B camera (Roper Scientific, Martinsried, Germany).

### Statistical Analysis

All results are reported as mean ± standard error (SEM). The data were tested for normality using the D'Agostino-Pearson normality test and for equality of variances using the F test [28]. If normality and equality of variances were not rejected at the 0.05 significance level, the group means were compared using the t-test. Otherwise, for non-normally distributed data and/or the data with unequal variances, we used the nonparametric Mann-Whitney U test. A p-value less than 0.05 was considered to be statistically significant. We used Graphpad Prism 5 for statistical analysis (GraphPad Software, Inc., La Jolla, CA).

## Results

### Jugular vein ligation results in venous stenosis *in vivo* with formation of collateral vessels

**Figure 1:**
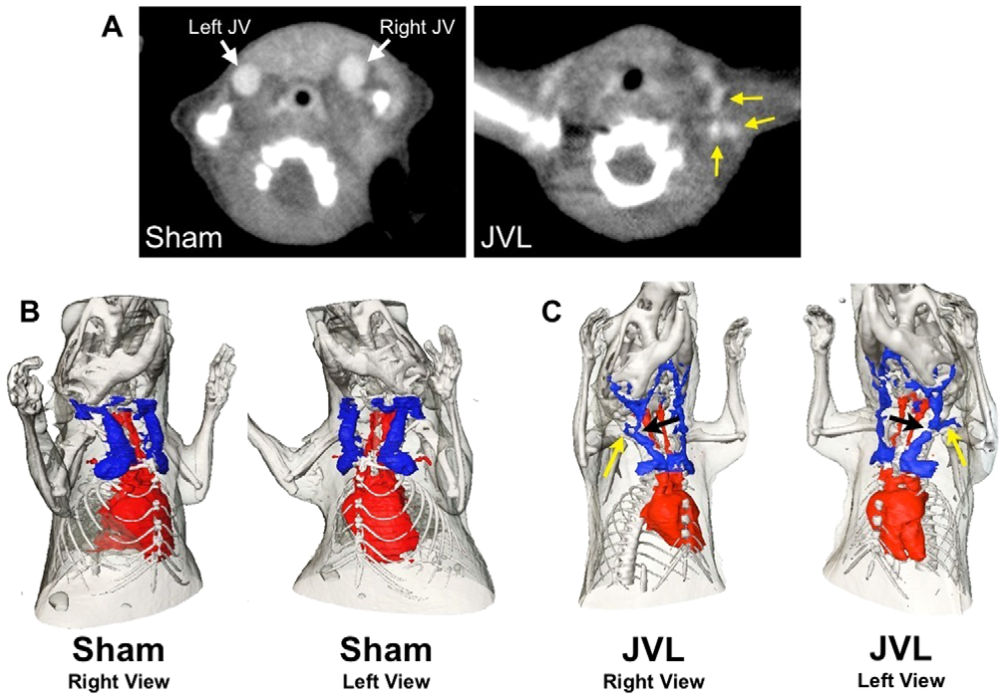
CT venogram and 3D vessel imaging of JV stenosis. (**A**) Sham mouse with patent JVs (white arrow). JVL mouse confirming ligation of the right and left JV and formation of collateral veins (yellow arrow). (**B**) Sham mouse exhibits normal venous drainage. (**C**) Right-side view of JVL mouse exhibits formation of small collateral vessels (yellow arrow) and stenosis due to surgical ligation (black arrow). Left-side view of JVL mouse exhibits complete occlusion at ligation site (black arrow) with formation of collateral vessels (yellow arrow).

Five months after surgery, CT venograms showed normal, widely patent jugular veins in sham mice (n = 3; Fig. 1A). In comparison, the CT venograms of the JVL group (n = 6) demonstrated severe stenosis of the jugular vein at the sites of ligation (Fig. 1A and B). In addition, there was small collateral vessel formation in the ligated group (yellow arrows in Fig. 1A and B). These results confirm that jugular vein ligation resulted in structural changes in the venous drainage.

### Jugular vein ligation alters cerebral hemodynamics

After confirming successful bilateral JV ligation, we investigated whether JVL resulted in altered cerebral hemodynamics. The JVL group demonstrated significantly increased cerebral retention of nuclear perfusion agent, Tc-99m-exametazime, compared to the sham group expressed as the percentage of the injected dose per gram of tissue (%IDGT = 7.09±0.42 (sham) *vs*. 8.29±0.11 (JVL), p = 0.024, Fig. S1) at 5 months post surgery. These results indicate that bilateral jugular vein ligation altered cerebral hemodynamics in the JVL group. Together with the structural abnormalities seen on CT venograms, these results confirm that we have established a mouse model of chronic cerebral venous insufficiency.

### Chronic cerebral venous insufficiency in mice does not result in blood-brain barrier breakdown or increased MMP activity

**Figure 2:**
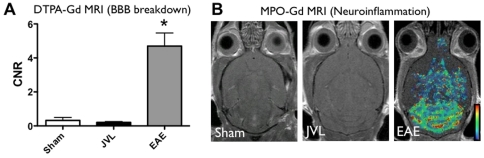
MR imaging to assess the blood-brain barrier and myeloperoxidase (MPO) activity. (**A**) MR imaging with DTPA-Gd does not reveal changes in blood-brain barrier permeability. Sham group (n = 3, CNR (contrast-to-noise ratio) = 0.32±0.17, JVL group (n = 6, CNR = 0.21±0.051, p = 0.58). EAE data provided for comparison (n = 3, 4.7±1.3, p = 0.024 vs. JVL). (**B**) MPO activity map from MPO-Gd MRI to detect areas of inflammation. A representative sham mouse (left) demonstrates no evidence of MPO activity. A representative JVL mouse (middle) demonstrates no evidence of MPO activity. A stage 2 EAE mouse (right) for comparison.

As expected, gadolinium-enhanced MR imaging using DTPA-Gd performed 4–6 months after surgery did not reveal any white matter lesions or abnormal enhancement in the sham group. However, we also did not detect any T2 hyperintense lesions or abnormal enhancement in the JVL group. Quantification of MRI data for increased parenchymal signal enhancement that would indicate leakage of the contrast agent across the BBB showed no statistically significant difference between the sham and JVL groups (Fig. 2A), demonstrating that the BBB remained intact.

**Figure 3:**
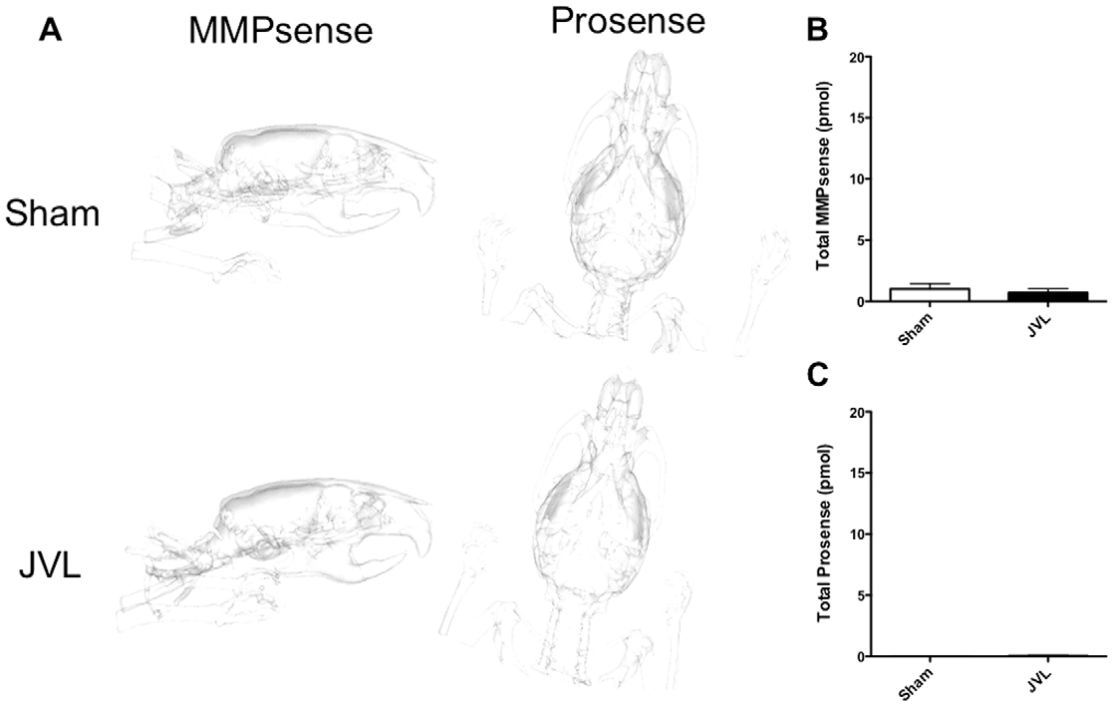
FMT-CT imaging of MMP and protease activity. (**A**) Representative images from sham and JVL mice show no visible signal from FMT imaging. (**B**) Quantification of the fluorescence signal demonstrates barely measurable signal and no significant difference between sham and JVL mice.

Furthermore, when we noninvasively assessed for MMP activity, which can be related to BBB breakdown [22], [23], we again found no significant difference between sham and JVL mice in the amount of the activated fluorescent probe detected, which was minimal and could not be visualized (Fig. 3A). As a reference, an inflammatory lesion would have 20–40 pmol of MMPsense activity [29].

We also examined for subtle break down in the BBB by assessing for the presence of the barrier molecules associated with an intact BBB (occludin and claudin-5, Fig. S2). Both sham and JVL mice sections demonstrated similar colocalization of these molecules with the endothelium (CD31), consistent with an intact BBB. On the other hand, EAE mice showed endothelial cells that were not associated with these barrier molecules, revealing that breakdown in the BBB had occurred.

### Chronic cerebral venous insufficiency in mice does not result in neuroinflammation or demyelination

We did not observe any evidence of increased MPO activity, and thus inflammation in vivo in either the sham or the JVL groups (Fig. 2B, compare to a representative EAE mouse with stage 2 disease). We also imaged for protease (cathepsins) activity on FMT-CT but again, did not find any significant difference between sham and JVL mice (Fig. 3A, C), which demonstrated minimal signal that could not be visualized (Fig. 3A). An inflammatory focus has been found to exhibit 20–40 pmol of activity [30].

**Figure 4:**
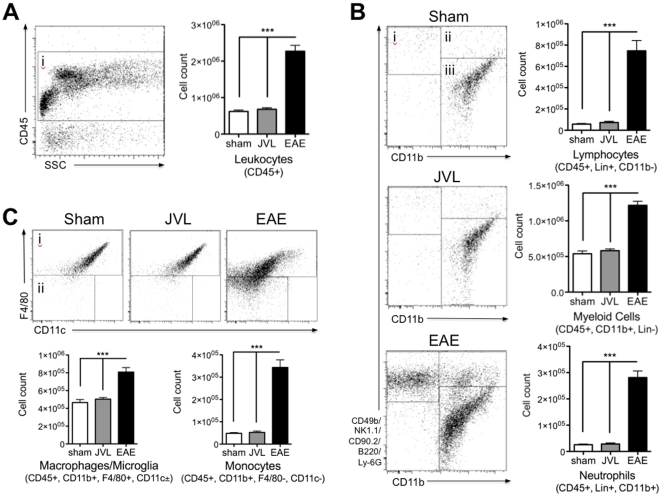
Flow cytometric analysis of brain inflammatory cells. (**A**) Cells were pre-gated for positive CD45 expression to identify all leukocytes, and (**B**) then divided into lymphocytes, neutrophils, and myeloid cells. The total number of all leukocytes, but also lymphocytes, neutrophils, and myeloid cells in the brain was unaffected in JVL mice (n = 4) compared to sham (n = 4), but significantly increased in EAE mice (n = 5). (**C**) Differentiating macrophages/microglia from monocytes showed that there were almost no monocytes in the brain of JVL and sham mice, but both cell types were increased to high numbers in EAE mice. Lin = CD90, NK1.1, B220, CD49b, and Ly-6G.

We also wanted to identify if JVL caused immune cell population shifts in the brain (Fig. 4). Similar to the imaging results, there was no significant change in the number of macrophages/microglia, monocytes, lymphocytes, and neutrophils in the brains of sham and JVL mice, though all of these cells are markedly increased in the brains of EAE mice. Together, these findings show that JVL did not result in increased neuroinflammation.

**Figure 5:**
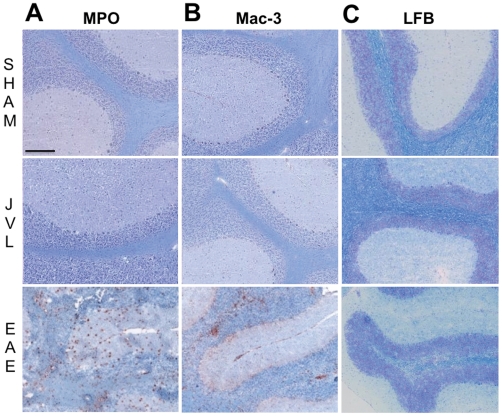
Histopathology of sham (top row), JVL, (middle row), and EAE (bottom row) groups. (**A**) MPO stain for MPO-positive cells/myeloid cells, (**B**) Mac-3 stain for activated macrophages/microglia, and (**C**) LFB stain for demyelination. Bar = 50 µm.

Histopathological assays on sham and JVL mice at 5 months post-ligation showed that both sham and JVL groups were devoid of MPO-positive cells (Fig. 5A). Furthermore, we did not observe macrophage/microglia infiltration, even though these are typically present in human MS plaques as well as experimental murine models of demyelinating disease (Fig. 5B). As the most important hallmark of MS is demyelination, we could not find any evidence of demyelination (Fig. 5C). Therefore, our results found no evidence of either inflammatory cellular infiltrate or products of inflammation in either the sham or the JVL groups to suggest neuroinflammation or demyelination at the time of sacrifice.

### Chronic cerebral venous insufficiency in mice does not result in clinical signs

**Figure 6:**
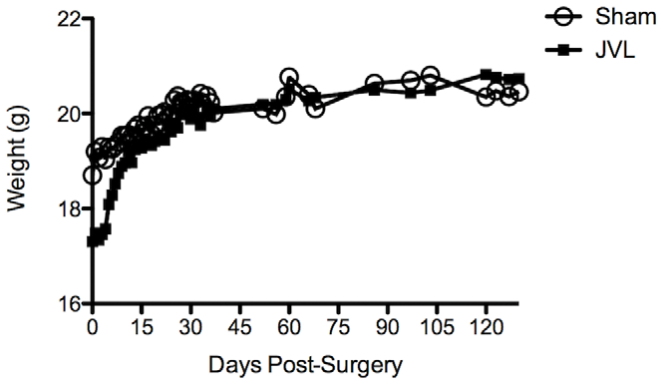
Weights of sham group (n = 6, open circle) and JVL group (n = 16, solid square) up to day 130 post-surgery.

Mice were also followed longitudinally (6 months) for clinical signs or disease progression using a 5-point EAE scoring system [16]. None of the mice in the JVL group or sham group showed any clinical signs (Fig. S3) throughout the duration of the study. Both groups exhibited a steady increase in weight and did not undergo the typical weight loss observed in diseased or impaired mice (Fig. 6).

## Discussion

In this study, we developed a murine model of chronic cerebral venous insufficiency in order to evaluate the relationship between CCSVI and demyelination. Our model consisted of bilateral JV ligation to mirror the cerebral venous abnormalities previously reported [6], including multiple stenoses, formation of the cervical collateral venous drainage pathways similar to those described in CCSVI [6], and cerebral hemodynamic disturbances. Despite both structural and hemodynamic abnormalities in the JVL group consistent with successful induction of chronic cerebral venous insufficiency, we did not find any evidence of BBB breakdown, neuroinflammation, or demyelination in the brain. For each parameter of disease progression that we assessed, we consistently found no difference between the sham and JVL groups. Our findings suggest that even significant disturbances in JV hemodynamics, such as those seen in this model, do not result in neuroinflammation and/or demyelinating plaques.

While animal models are invaluable research tools in helping us understand human disease, they are imperfect models. The human circulation system differs from that of the mouse in both structure and position. Since humans are bipeds and stand erect, the human brain axis is aligned approximately 90 degrees more ventral than the mouse brain with respect to the axis of the body. The exact effect that this postural difference has, if any, on mouse cerebral hemodynamics versus human cerebral hemodynamics is unclear. Furthermore, it is possible that additional stress or stimuli might be needed to increase cardiovascular demand in our animal model since mice are more sedentary compared to humans. Both posture and activity may therefore potentially play a role in exacerbating the effects of venous congestion resulting from venous stenosis. To address these possible confounding variables, we aimed to maximize the resting state of venous pressure by surgically ligating both JVs in order to create an extreme scenario of increased venous cerebral venous insufficiency. Some studies have reported that 91% of MS patients having either unilateral or bilateral JV stenosis, with 14% exhibiting bilateral stenosis in the jugular veins [6]. In this way, we believe we have accounted for possible differences in cerebral venous hemodynamics between mice and humans by creating a murine model with relatively greater cerebral venous insufficiency than previous positive studies have reported in humans [6]. The mice were followed for up to 6 months after JVL, a considerable part of the lifespan of SJL mice (typically around 400 days) which, particularly given the maximization of venous pressure by bilateral JVL, should be more than sufficient for disease manifestation if there was a relationship between venous congestion and demyelination.

Currently, the hypothesis that CCSVI causes MS is unproven but remains highly attractive to many patients and physicians. Conflicting results have been found in clinical trials. Our longitudinal animal study evaluated for multiple fundamental signs known to occur in MS in order to assess for a relationship between chronic venous congestion and demyelination. We found that chronic cerebral venous insufficiency in our animal model does not cause BBB breakdown, neuroinflammation, demyelinating plaques, or clinical manifestations that are the hallmarks of a demyelinating disease such as multiple sclerosis. Therefore, our animal study does not support chronic cerebral venous insufficiency as a cause of multiple sclerosis.

## Supporting Information

Figure S1 **Gamma scintillation counter using Tc-99m-exametazime.** Sham group (n = 4, %IDGT (% injected dose per gram of tissue) = 7.09±0.42), JVL group (n = 4, %IDGT = 8.29±0.11), p = 0.024.
(DOCX)

Figure S2 **Double immunofluorescence to assess barrier molecules.** (A) Claudin-5 and CD31 in jugular vein ligation mice compared with sham and EAE mice. In areas of neuroinflammation in EAE, as indicated by MPO immunohistochemistry in adjacent sections (top row), there is a general paucity of the barrier molecule claudin-5 (red), and there is a loss of co-staining of claudin-5 with the endothelial cell marker CD31 (green, arrows). In jugular vein ligation and sham animals there is no change in the staining pattern for claudin-5, and all vessels show co-staining for CD31, demonstrating no alteration of the blood-brain barrier. Cell nuclei are stained with DAPI (blue). Black rectangles in top row show areas shown for immunofluorescence. Similar findings were found for (B) occludin and CD31.
(DOCX)

Figure S3 **EAE clinical scores of sham group (n = 6, open circle) and JVL group (n = 16, closed square) up to day 130 post-surgery.**
(DOCX)
