# Supplementary material for: Ligation of the Jugular Veins Does Not Result in Brain Inflammation or Demyelination in Mice
Source: PLoS One. 2012 Mar 21;7(3):e33671. doi: 10.1371/journal.pone.0033671 (PMC3310075; doi:10.1371/journal.pone.0033671)
Supplement: Figure S1 — Gamma scintillation counter using Tc-99m-exametazime. Sham group (n = 4, %IDGT (% injected dose per gram of tissue) = 7.09±0.42), JVL group (n = 4, %IDGT = 8.29±0.11), p = 0.024. (DOCX) [file pone.0033671.s001.docx]

Ligation of the Jugular Veins Does Not Result in Brain Inflammation or Demyelination in Mice

Wendy Atkinson, BS^1,*^, Reza Forghani, MD, PhD^1,2,4,*^, Gregory R. Wojtkiewicz, MS^1^, Benjamin Pulli, MD^1^, Yoshiko Iwamoto, BS^1^, Takuya Ueno, MD, PhD^1^, Peter Waterman, MBA^1^, Jessica Truelove, BS^1^, Rahmi Oklu, MD, PhD^3^, and John W. Chen, MD, PhD^1,2^

^1^Center for Systems Biology, Massachusetts General Hospital and Harvard Medical School, Richard B. Simches Research Center, 185 Cambridge Street, Suite 5.210, Boston, MA, USA 02114

^2^Division of Neuroradiology, Department of Radiology, Massachusetts General Hospital and Harvard Medical School, 55 Fruit St., GRB-285, Boston, MA USA 02114

^3^Division of Vascular Imaging & Intervention, Department of Radiology, Massachusetts General Hospital and Harvard Medical School, 55 Fruit St., GRB-290A, Boston, MA USA 02114

^4^Sir Mortimer B. Davis Jewish General Hospital and McGill University, Room C-210.2, 3755 Cote Ste-Catherine Rd, Montreal, Quebec, Canada H3T 1E2

*WA and RF contributed equally.

Fig. S1: Gamma scintillation counter using Tc-99m-exametazime. Sham group (n=4, %IDGT (% injected dose per gram of tissue)=7.09 ± 0.42), JVL group (n=4, %IDGT=8.29 ± 0.11), p=0.024.
