# Supplementary material for: Ligation of the Jugular Veins Does Not Result in Brain Inflammation or Demyelination in Mice
Source: PLoS One. 2012 Mar 21;7(3):e33671. doi: 10.1371/journal.pone.0033671 (PMC3310075; doi:10.1371/journal.pone.0033671)
Supplement: Figure S2 — Double immunofluorescence to assess barrier molecules. (A) Claudin-5 and CD31 in jugular vein ligation mice compared with sham and EAE mice. In areas of neuroinflammation in EAE, as indicated by MPO immunohistochemistry in adjacent sections (top row), there is a general paucity of the barrier molecule claudin-5 (red), and there is a loss of co-staining of claudin-5 with the endothelial cell marker CD31 (green, arrows). In jugular vein ligation and sham animals there is no change in the staining pattern for claudin-5, and all vessels show co-staining for CD31, demonstrating no alteration of the blood-brain barrier. Cell nuclei are stained with DAPI (blue). Black rectangles in top row show areas shown for immunofluorescence. Similar findings were found for (B) occludin and CD31. (DOCX) [file pone.0033671.s002.docx]

Ligation of the Jugular Veins Does Not Result in Brain Inflammation or Demyelination in Mice

Wendy Atkinson, BS^1,*^, Reza Forghani, MD, PhD^1,2,4,*^, Gregory R. Wojtkiewicz, MS^1^, Benjamin Pulli, MD^1^, Yoshiko Iwamoto, BS^1^, Takuya Ueno, MD, PhD^1^, Peter Waterman, MBA^1^, Jessica Truelove, BS^1^, Rahmi Oklu, MD, PhD^3^, and John W. Chen, MD, PhD^1,2^

^1^Center for Systems Biology, Massachusetts General Hospital and Harvard Medical School, Richard B. Simches Research Center, 185 Cambridge Street, Suite 5.210, Boston, MA, USA 02114

^2^Division of Neuroradiology, Department of Radiology, Massachusetts General Hospital and Harvard Medical School, 55 Fruit St., GRB-285, Boston, MA USA 02114

^3^Division of Vascular Imaging & Intervention, Department of Radiology, Massachusetts General Hospital and Harvard Medical School, 55 Fruit St., GRB-290A, Boston, MA USA 02114

^4^Sir Mortimer B. Davis Jewish General Hospital and McGill University, Room C-210.2, 3755 Cote Ste-Catherine Rd, Montreal, Quebec, Canada H3T 1E2

*WA and RF contributed equally.

**A**

**B**

**Fig S2:** Double immunofluorescence to assess barrier molecules. (A) Claudin-5 and CD31 in jugular vein ligation mice compared with sham and EAE mice. In areas of neuroinflammation in EAE, as indicated by MPO immunohistochemistry in adjacent sections (top row), there is a general paucity of the barrier molecule claudin-5 (red), and there is a loss of co-staining of claudin-5 with the endothelial cell marker CD31 (green, arrows). In jugular vein ligation and sham animals there is no change in the staining pattern for claudin-5, and all vessels show co-staining for CD31, demonstrating no alteration of the blood-brain barrier. Cell nuclei are stained with DAPI (blue). Black rectangles in top row show areas shown for immunofluorescence. Similar findings were found for (B) occludin and CD31.
